# Elastic fibres in alcoholic liver disease

**DOI:** 10.1038/s41598-020-77007-z

**Published:** 2020-11-18

**Authors:** Tu Vinh Luong, Sameh Abou-Beih, Jennifer Watkins, Emmanuel Tsochatzis, Massimo Pinzani, Stephen Davison, Andrew Hall, Alberto Quaglia

**Affiliations:** 1 Department of Cellular Pathology, UCL Cancer Institute, Research Department of Pathology, Royal Free London, Pond Street, London, NW3 2QG UK; 2 UCL Institute for Liver and Digestive Health, Royal Free Hospital and UCL, London, UK; 3 Pathology Department, Faculty of Medicine, Fayoum University, Fayoum, Egypt

**Keywords:** Hepatology, Pathogenesis, Inflammation

## Abstract

The literature on the contribution of elastic fibre deposition to alcohol-related liver disease (ARLD) is limited. We studied: (1) 180 liver biopsies from ARLD patients; (2) 20 ARLD explant livers; (3) 213 liver biopsies with non-ARLD injury. Elastic fibres were assessed in terms of their distribution around hepatocytes [pericellular elastosis (PCE)] and within bridging fibrous septa (septal elastosis) and scored using a semiquantitative system. We also investigated the composition of the elastic fibres (oxytalan, elaunin and mature elastic fibres) in 20 cases. PCE was associated with steatohepatitis in ARLD patients and with ARLD when compared to non-ARLD cases (p < 0.001). Oxytalan fibres were identified in PCE in ARLD biopsies and broken dense perisinusoidal mature elastic fibres in explanted livers. Septal elastosis increased from intermediate to advanced fibrosis stage. Early septal elastosis contained oxytalan fibres, whereas septal elastosis at more advanced stages contained mainly mature elastic fibres. PCE is a typical feature of steatohepatitis in ARLD and includes oxytalan fibres. Septal elastosis is a gradual process with a transition from oxytalan to mature elastic fibres usually present in explanted livers. There may be different dynamics in the assembly and reabsorption of pericellular and septal elastic fibres, and a potential role for stratification of patients with advanced stage ARLD.

## Introduction

Worldwide alcohol-related mortality in 2016 was high at 38.8 per 100,000 people and half of cirrhosis related deaths were due, at least in part, to alcohol use^1^. The spectrum of liver injury in alcohol-related liver disease (ARLD) ranges from simple steatosis to steatohepatitis and from minimal or no fibrosis to cirrhosis^2^. Elastic fibres are a component of the extracellular matrix (ECM) along with collagen, glycoproteins, glycosaminoglycans and proteoglycans^3^. The elastic fibres are composed of a core of elastin made of tropoelastin monomers encoded by *ELN* and a microfibrillar mantle composed of fibrillins, glycoproteins which in humans are present in three isoforms^3,4^. The relative proportion of tropoelastin and microfibrils divide elastic fibres into three main types: oxytalan, composed of microfibrils only with no elastin core; and elaunin and mature elastic fibres, both characterized by a central cross-linked core of elastin and a surrounding microfibrillar component. Elaunin fibres have a lower amount of elastin compared to mature elastic fibres^5^. Oxytalan fibres can be differentiated from both elaunin and mature elastic fibres by removing the oxidation step of histochemical elastic fibre staining methods such as the orcein stain or analogues^6–9^. Oxytalan fibres do not react with this modified protocol, whereas both elaunin and mature elastic fibres remain positive. Elastogenesis begins in the late stages of development and early after birth^10^ and is particularly active in those tissues in which elastic properties are essential for their function such as the aortic wall and lung parenchyma^4^. Elastogenesis is attenuated in normal adult tissues but reactivates in a wide spectrum of disease processes^10^ including wound healing^11,12^. Elastin has long been known to accumulate in fibrotic livers^4,13^. Little is known however about its role in the progression of ARLD and the literature on the contribution of elastic fibre deposition in ARLD injury is limited^14^. Porto et al.^14^ showed that in the early stage of alcoholic fibrosis there is deposition of oxytalan fibres in the space of Disse in the perivenular region. The internodular bridging fibrous septa of more advanced stages contained oxytalan and elaunin fibres. Our aim was to evaluate retrospectively a large series of liver biopsies from patients with ARLD focusing on the pattern, distribution and composition of elastic fibres.

## Results

Out of our initial cohort of 303 biopsies 180 fulfilled our selection criteria and were selected for our study.

### Overall pattern of injury and disease stage

Seventy-three biopsies (40.6%) showed steatosis only (mild in 36 (49.3%), moderate in 28 (38.4%) and severe in 9 (12.3%). Ninety-seven biopsies (53.9%) showed steatohepatitis. Ten (5.6%) biopsies were classified as NOS (Supplementary Figure 1). According to the limited clinical history available at the time of this retrospective review, these ten patients with NOS changes were patients usually biopsied to ascertain fibrosis stage in the context of suspected or known ARLD. One of these patients had been abstinent at the time of the liver biopsy.

The frequency (and percentage) of liver biopsies at the different disease stage were as follows: 0 (no fibrosis) = 4 (2.2%); 1 (early) = 84 (46.7%); 2 (intermediate) = 34 (18.9%); 3 (advanced stage cirrhotic transformation) = 13 (7.2%); 4 (advanced stage cirrhotic) = 45 (25%).

### Pericellular elastic fibres

**Figure 1:**
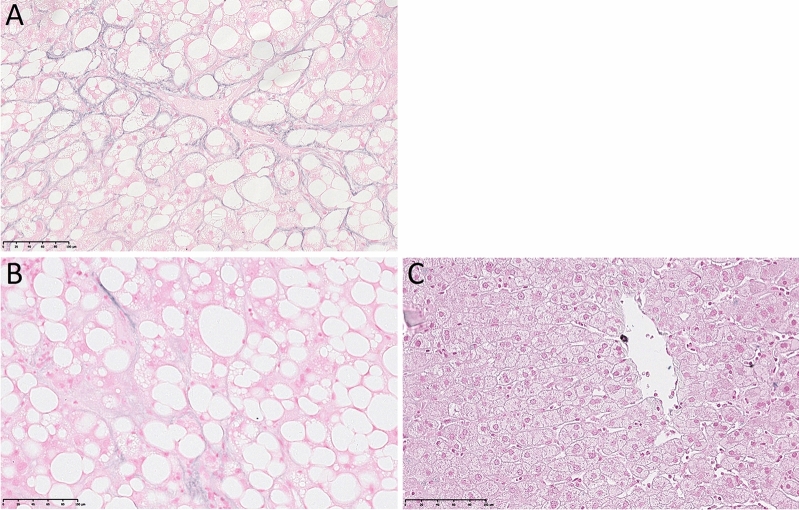
Victoria blue stain. Pericellular elastosis (**A**); focal pericellular elastic fibres (**B**); no pericellular elastic fibres in normal liver (**C**).

For reference, examples of none and focal perihepatocytic elastic fibres, and PCE are demonstrated in Fig. 1.

The correlation between pericellular elastic fibres and overall disease pattern is detailed in Supplementary Table 1.

**Figure 2:**
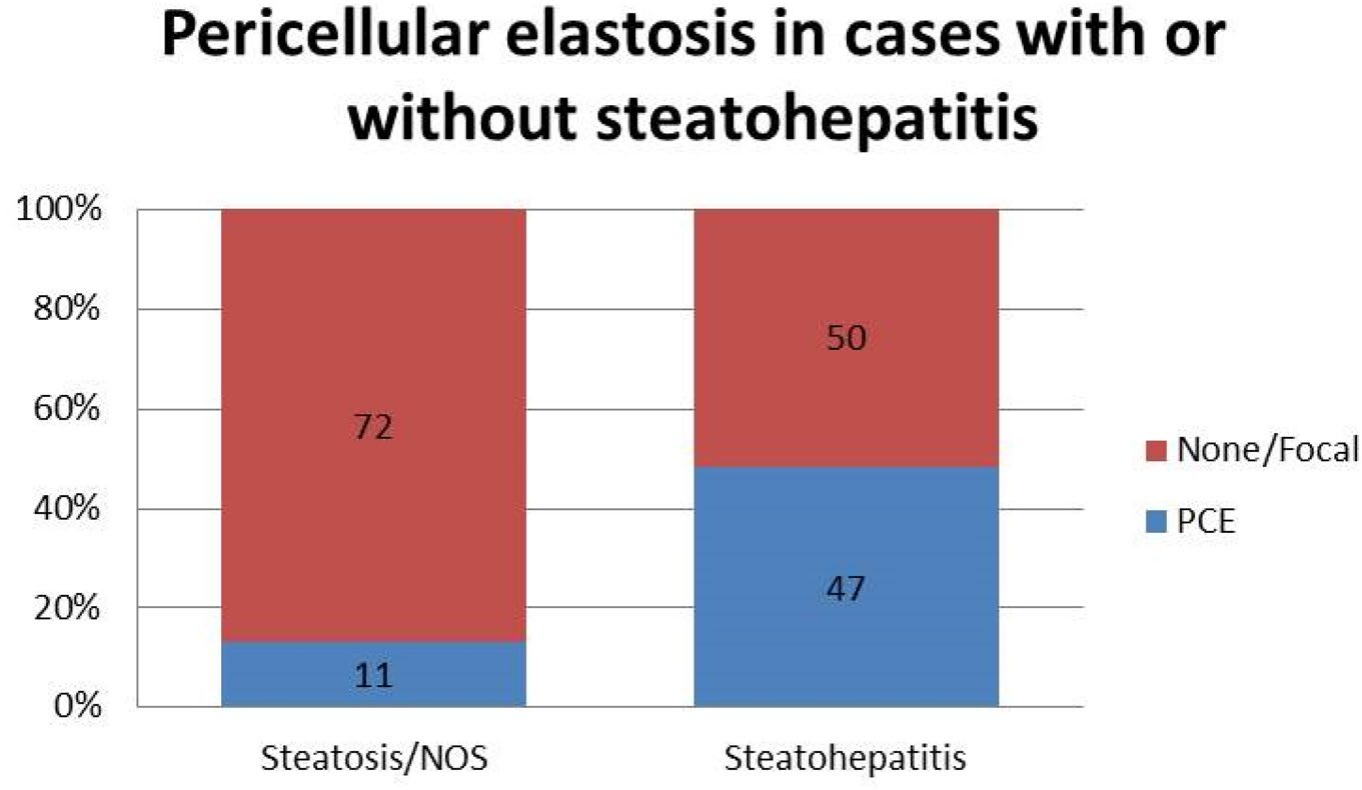
Pericellular elastosis in cases of steato hepatitis compared to cases without steato hepatitis.

PCE was identified in 58 (32.2%) of the 180 cases studied. PCE was present in 7 (9.6%) of the 73 biopsies with steatosis, 47 (48.5%) of the 97 biopsies with steatohepatitis, and 4 (40%) of the 10 biopsies classified as NOS. When biopsies were divided into two groups according to presence or absence of steatohepatitis, PCE was significantly associated with steatohepatitis (p < 0.001, Fig. 2).

### Control cohort

As shown in Supplementary Table 2, our comparison with the control cohort of 213 liver specimens showed that PCE is rare in conditions other than ARLD with the exception of non-alcoholic steatohepatitis (NASH), and vascular disorders in which it was observed in 30% and 55.6% of cases respectively. The difference between these control groups and our ARLD biopsy study cohort reached statistical significance (p < 0.001, not shown).

### Septal elastic fibres

**Figure 3:**
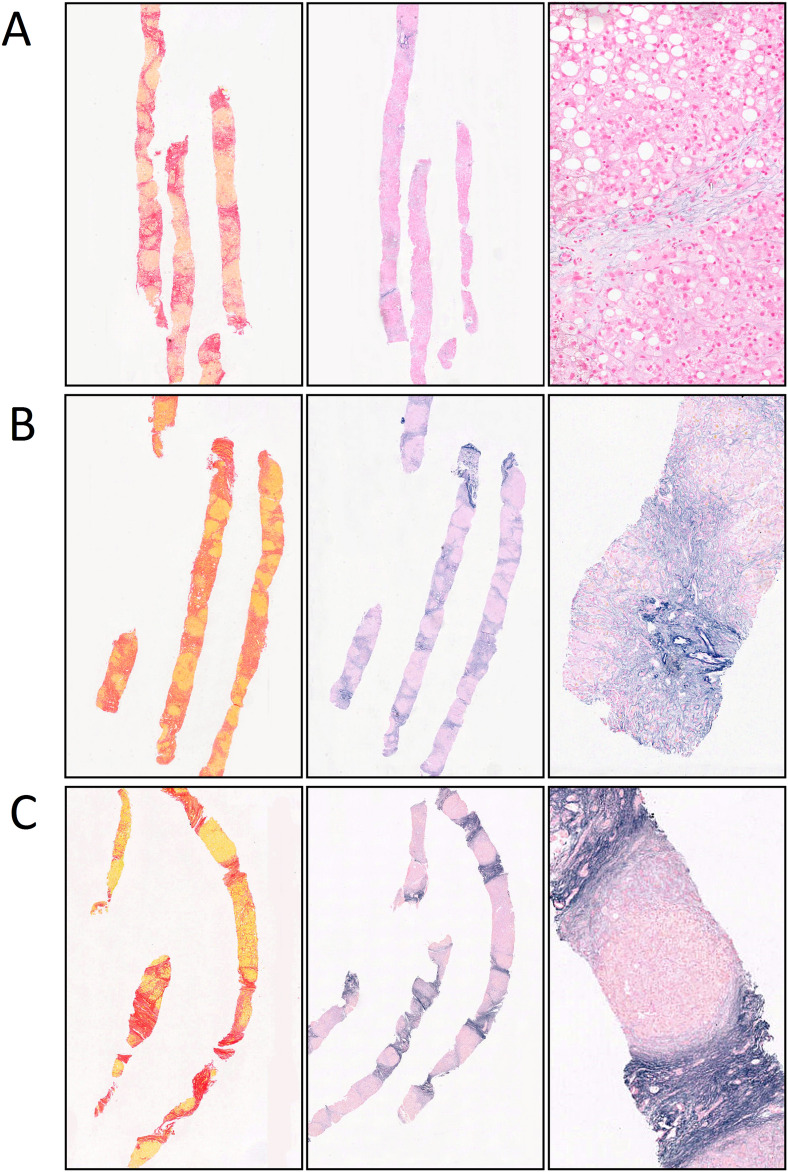
Elastic fibres grades. (**A**) Elastic fibres grade 1: (bridging fibrous septa with delicate fibres visible only at high magnification) in a stage 4, advanced stage cirrhotic. (**B**) Elastic fibres grade 2: (elastic fibres visible at low magnification and clearly distinguishable from the residual portal elastic fibres) in a stage 4, advanced stage cirrhotic. (**C**) Elastic fibres grade 3: (strong elastic bundles well visible at low magnification and of similar density, blending with or indistinguishable from the to the residual portal normal elastic fibres) in a stage 4, advanced stage cirrhotic.

**Figure 4:**
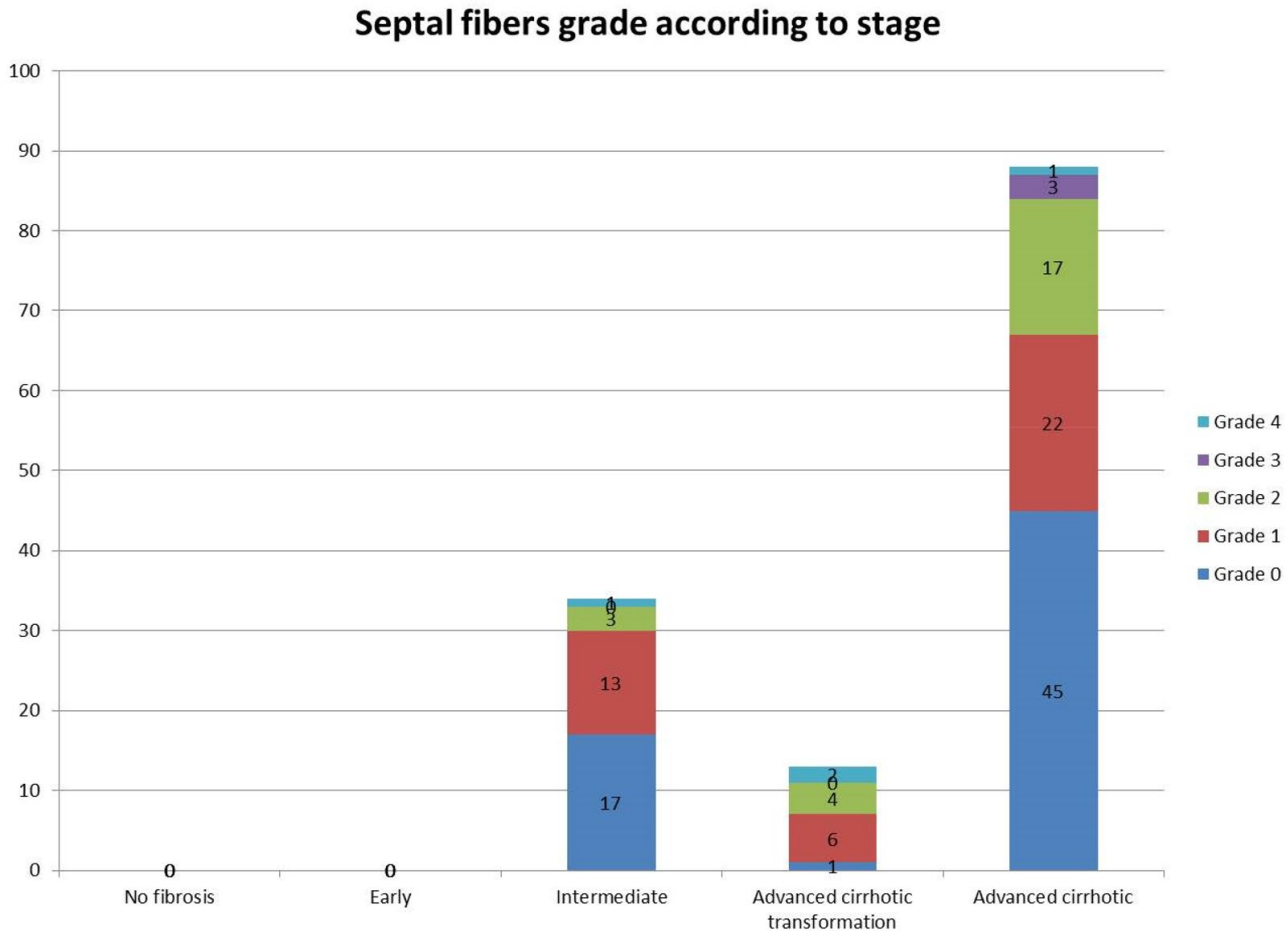
Septal elastic fibre grades according to fibrosis stage.

The categorization of septal fibres is demonstrated in Fig. 3. The distribution of the septal elastic grades is detailed in Supplementary Table 3 and illustrated in Fig. 4.

There was an increase in the proportion of cases exhibiting septal grade 1, 2 and 3 in advanced disease stage compared to intermediate disease stage; this corresponded to a decrease in grade 0. Grade 4, regressive type, was uncommon and appeared to be consistent amongst disease stages.

### End stage cohort: elastic fibres in livers removed at transplantation for alcohol-related cirrhosis

These 20 livers were removed at transplantation from 20 patients (15 males, 5 females, age range 38–64 year old, median 54 years) with end stage ARLD. All patients had been abstinent at the time of transplantation for at least 6 months.

Most cases showed a combination of grades 2, 3 and 4 of septal elastic fibres. Grade 0 was not observed in any of these 20 cases and grade 1 was present in three cases only. PCE was not identified in most cirrhotic nodules in 18 cases. It was present very focally in occasional cirrhotic nodules after extensive examination at high magnification in 4 cases, and was easily identified at low magnification in a few (7 cases) or several (7 cases) cirrhotic nodules. The pericellular elastic fibres were often in the form of broken dense strands or in a more perisinusoidal distribution. Examples are shown in Supplementary Figure 2.

In two other cases PCE was more prominent affecting approximately half of the cirrhotic nodules in the sample examined. There was no evidence of active steatohepatitis.

### Mixed ARLD sub-series: morphology and composition of elastic fibres

We next correlated our findings with the immunohistochemistry for elastin and the repeat Victoria blue stain with and without the oxidation step to explore the proportion of oxytalan, elaunin and mature elastic fibres. (Supplementary Table 4 and Supplementary Figures 3, 4).

In terms of septal elastic fibres (not shown), in all ten biopsies showing grade 1 or grade 2 septal elastosis with the conventional Victoria blue an appreciable reduction in elastic fibre stain with the modified Victoria blue method suggested the presence of a significant proportion of oxytalan.

Grade 3 or 4 septal elastosis, consisted of elastin positive septa without reduction in stain with the modified Victoria blue stain indicating that the septa were composed mostly of mature elastic fibres.

PCE appears to be composed of a significant proportion of oxytalan in most of the biopsy specimens and of mature elastin in the explanted livers (Supplementary Figures 3 and 4).

## Discussion

Physiological during fetal development and early after birth, elastogenesis reactivates in many disease processes including liver disorders^4^. The literature on elastogenesis in ARLD is scanty. Porto et al.^14^ showed that the deposition of oxytalan fibres occurs at an early stage in the space of Disse in the perivenular region, and elaunin fibres accumulate in septa at later stages.

In our study PCE was significantly associated with the presence of steatohepatitis regardless of fibrosis stage. In contrast, PCE was rare in the ARLD biopsies with steatosis, or non-specific changes and was either absent or focal in most liver explants, in which broken dense strands often in perisinusoidal rather than pericellular distribution were present. Our additional histochemical analysis indicates that PCE in biopsies includes a significant proportion of oxytalan fibres, in contrast to the residual mature elastic fibres present focally in explanted livers and in keeping with PCE remnants after abstinence. Our findings therefore suggest that PCE is a dynamic process; retains a degree of reversibility even at an advanced disease stage; and that the morphology and composition of the elastic fibres may help in gauging disease activity Our study was based on serial sections, and we could not confirm whether PCE always co-localise with pericellular fibrosis. As elastic fibers are not identifiable in the space of Disse in normal conditions, the presence of PCE could be of help in confirming pericellular injury in those cases in which the increase in space of Disse collagen is subtle based on conventional connective tissue stains.

Abstinence from alcohol could result in the reabsorption of digestible oxytalan fibres, or slow the conversion of oxytalan into elaunin and mature elastin. Either or both processes combined with hepatocyte regeneration would explain the pattern of broken dense mature elastic fibres often oriented along the sinusoidal axis rather than enveloping individual hepatocytes. The presence of focal mature elastic fibres in biopsies with NOS changes or just steatosis could therefore represent remnants of previous episodes of steatohepatitis.

We observed that PCE is rarely present in other liver disorders with the exception of NASH and vascular disorders. In NASH the pattern of PCE is similar to ARLD. Perivenular elastic fibres were described by Nakayama et al.^15^, using the orcein stain in NASH with bridging fibrosis but not in earlier stages. Our comparison group included 30 cases of NASH, which showed that PCE can be present in early stage and absent in advanced stage NASH, but a larger series of cases would be necessary for further correlations and considerations.

The occurrence of PCE in ARLD, NASH and vascular disorders fits with their pathogenesis based on an insult transmitted directly to the space of Disse. Fibrillin-1 is present physiologically in the space of Disse and forms a network between hepatic stellate cells and hepatocytes in the absence of elastin. Fibrillin-1 could act as the scaffold for the assembly of tropoelastin into coacervates leading to the formation eventually of mature elastic fibres^4,16^.

In line with the findings by Porto and colleagues^14^, the deposition of elastic fibres in bridging fibrous septa seems to occur gradually starting as delicate strands visible only at higher magnification, and made initially of oxytalan, and progressing to the dense strong elastic bundles made of mature elastic fibres, and usually observed at the advanced/end disease stage. Yasui et al.^17^ showed recently that in chronic hepatitis C the quantity of elastic fibres increases significantly from fibrosis stage 2 to stage 3. Our findings therefore could be helpful in stratifying patients with advanced disease stage, and who could not be differentiated further based on conventional fibrosis scoring system or with image analysis method such as CPA^18^. Further work is needed to investigate how elastic fibre deposition correlate with conventional fibrosis staging and clinical outcome.

Whether the grade of septal elastosis helps in gauging disease reversibility however remains uncertain. A longitudinal study by Bedossa et al.^19^ on liver biopsies from patients treated with alpha-interferon for chronic viral hepatitis showed that elastic fibres could not be detected in portal tracts during the active phase of the disease. Healing was associated with the deposition of elastic fibres initially as long thin and parallel fibres, and later as thicker and tortuous fibres wrapped around thick bundles of collagen. It is therefore possible that the elastification of septa is dependent on disease duration, and represents a marker of chronicity, rather than a threshold of clinico-pathological reversibility. Alcohol withdrawal may not necessarily stop the elastification process, which would explain the prevalence of grades 3 and 4 in explanted livers even after prolonged periods of abstinence. Grade 4 in particular, in the form of slender dense elastic bundles corresponding to the known slender fibrous septa of the hepatic repair complex described by Wanless^20,21^, could be the result of partial reabsorption and thinning of broader fully elastified and collagenized septa or reabsorption of collagen followed later by complete elastification as part of the healing process. Nevertheless, the presence of mature elastic fibres in slender septa could help in differentiating between early bridging and regressed fibrosis.

In conclusion, the benefit of our study is that it provides an insight into how the elastic component of the extracellular matrix may change during the course of ARLD. PCE is associated with histological steatohepatitis and the pattern of deposition of elastic fibres in bridging fibrous septa is a gradual process. Different dynamics in the assembly and reabsorption of pericellular and septal elastic fibres could be the reason behind the opposite response to abstinence (i.e. disappearance of PCE and consolidation of septal elastic fibres). Further studies are needed to better understand whether the elastification process can be used to stratify patients with advanced stage ARLD and correlate with clinical outcome.

## Material and methods

Our study was based on four cohorts of specimens; (i) a series of 303 core needle biopsy specimens from patients with known ARLD clinically; (ii) a series of 20 livers removed at transplantation for ARLD; (iii) a control group of 213 core needle biopsy specimens with a variety of liver conditions other than ARLD characterized after clinico-pathological correlation; (iv) a selection of representative cases from cohorts (i) and (ii) to investigate in more details the elastic fibres morphology and composition in terms of oxytalan, elaunin and elastin content.

Our review of the first cohort of biopsies included the assessment of the overall pattern of liver injury, fibrosis stage, and pattern of elastic fibre deposition. The biopsies were divided into the following categories^16^: steatosis, steatohepatitis, and not otherwise specified (NOS).

Fibrosis staging was carried out using a semiquantitative scoring system (0 no fibrosis identified); 1 or early; 2 or intermediate (bridging fibrosis); 3 or advanced stage with features of cirrhotic transformation; and 4 or advanced stage cirrhotic.

We assessed the presence of elastic fibres in a pericellular/perivenular distribution and within fibrous septa.

Pericellular elastic fibres were classified as absent, focal (very occasional delicate perihepatocyte strands visible only at high magnification (400 × magnification) and after extensive search), or pericellular elastosis (PCE) (perihepatocytic elastic strands obvious at 10 × or 20 × magnification).

We classified septal elastosis into the following grades: grade 0: bridging fibrous septa with no elastic fibres; grade 1: bridging fibrous septa with delicate fibres visible only at high magnification; grade 2: elastic fibres visible at low magnification and clearly distinguishable from the residual portal or liver capsule elastic fibres, grade 3: strong elastic bundles well visible at low magnification and of similar density, blending with or indistinguishable from the to the residual portal normal or liver capsule elastic fibres; and grade 4: slender, regressive type dense elastic bundles visible at low magnification. Pericellular and septal elastosis were further investigated, in terms of their composition on a subseries of 20 cases using 6 serial sections. The sections were stained in the following sequential order with (i) anti-Elastin [BA4] antibody IHC (ab9519), (ii) Victoria blue with no oxidation step (demonstrating only mature elastin fibres and Elaunin), (iii) Victoria blue with oxidation and decolourisation step (demonstrating oxytalan in addition to elaunin and mature elastin), (iv) (spare unstained back-up section), (v) Picrosirius red and (vi) Haematoxylin and Eosin. Reduction in stain between the Victoria blue with and without oxidation step was assessed as follows: minimal and focal (reduction present focally after extensive comparison of the two staining methods); partial (easily identified and in approximately one/two thirds of septa or pericellular areas, with a significant proportion of areas with elastic fibres retaining the stain); marked (easily identified and in more than two thirds of, or all septa or pericellular areas). Septal elastin immunohistochemical stain was regarded as diffuse when present in most or all septa and patchy when approximately half of the septa were negative. Pericellular elastin stain was considered as diffuse (most hepatic plates), patchy (positive in approximately half), focal (identified at high magnification after extensive search) or absent.

Details on the biopsy selection, histological categories, fibrosis scoring system staining protocols, statistical analysis and elastic fibres assessment are provided as supporting information. All methods were carried out in accordance with relevant guidelines and regulations, experimental protocols were approved by the Royal Free Hospital Ethics Committee (07/Q0501/50) and informed consent was obtained as appropriate.

## Supplementary information

Supplementary Information.
