# Supplementary material for: Elastic fibres in alcoholic liver disease
Source: Sci Rep. 2020 Nov 18;10:20098. doi: 10.1038/s41598-020-77007-z (PMC7674436; doi:10.1038/s41598-020-77007-z)
Supplement: Supplementary file 1 — Supplementary Information. [file 41598_2020_77007_MOESM1_ESM.docx]

**Elastic fibres in alcoholic liver disease**

**Tu Vinh Luong^1^, Sameh Abou-Beih^1,3^, Jennifer Watkins^1^, Emmanuel Tsochatzis^2^, Massimo Pinzani^2^, Stephen Davison^1^, Andrew Hall^1^, and Alberto Quaglia^1^**

**1 Department of Cellular Pathology, Royal Free London**

**2 UCL Institute for Liver and Digestive Health, Royal Free Hospital and UCL, London**

**3 Pathology Department, Faculty of Medicine, Fayoum University**

**Correspondence to**

**Prof Alberto Quaglia**

**Department of Cellular Pathology**

**UCL Cancer Institute, Research Department of Pathology**

**Royal Free London**

**Pond Street**

**NW3 2QG**

**alberto.quaglia@nhs.net**

**Conflict of interest statement**

**The Authors do not have any competing interests in relation to the work presented in this manuscript.**

**Supporting information**

**Material and methods**

**ALD core needle biopsy specimens cohort**

This cohort consisted of 303 formalin fixed paraffin embedded liver biopsy specimens in the Royal Free Hospital archives between 1991 and 2016 from patients with a known clinical history of alcohol consumption to excess. We performed a preliminary review of this cohort to exclude inadequate samples due to their small size and/or fragmentation, significant concomitant pathology of other type (e.g. a marked portal lympho-plasmacytic infiltrate well in excess of what usually observed in patients with ALD, florid granulomatous inflammation, venous outflow block or signs of biliary disease etc.), or lack of relevant stains. The routine set of histochemical stains carried out in our laboratory included orcein and from approximately 1995 onwards Victoria blue as well. We decided to use only the Victoria blue stain for our study as our impression from the preliminary review was that it was more consistent than the orcein, resisted well to fading, and the blue color of the stain provided a sharper contrast and was easier to recognize particularly in the identification of the more delicate elastic strands. Portal, venular and/or liver capsule elastic fibres were used as internal positive control to verify that the stain was of sufficient quality.

The following criteria were used:-Steatosis (simple steatosis affecting more than 5% of hepatocytes, large or small droplet type and divided into mild (< 30% of hepatocytes) moderate (30-60% of hepatocytes) and severe (>60% of hepatocytes);

-Steatohepatitis (based on the presence of hepatocyte ballooning, with or without and infiltrate of neutrophils in the hepatic plates, with or without Mallory-Denk bodies). .

-Not otherwise specified (NOS). Those cases that did not fall into any of the above categories and did not show some of the other patterns of injury sometimes observed in ALD patients (e.g. alcoholic foamy degeneration) were classified as NOS.

**Fibrosis staging**

Fibrosis staging was carried out using a 0 to 4 scale based on the following considerations: 1) the categories used in the Clinical Research Network (CRN) [2] scoring system for non-alcoholic steatohepatitis were taken into account in view of the similarities in terms of fibrosis distribution between alcoholic and non-alcoholic steatohepatitis; 2) a compromise between the granularity of a complex scoring system like Ishak’s [3] to include a category intermediate between bridging fibrosis and cirrhosis (stage 5 or incomplete cirrhosis in the Ishak’s scoring system) and the reproducibility of a simpler one; 3) instances of advanced pericellular and bridging fibrosis with initial micronodular transformation, observed at times in biopsies from alcoholic patients and falling somewhere in between scores 3 and score 4 of the CRN system. We therefore designed and applied the following scoring system : 0 (no fibrosis identified); 1 or early (mild portal or periportal and/or perivenular sinusoidal fibrosis, but no bridging; 2 or intermediate (bridging fibrosis); 3 or advanced with features of cirrhotic transformation (parts of parenchymal nodules present in the biopsy sample or small nodules present in part of the biopsy sample); and 4 or advanced stage cirrhotic (clearly nodular parenchyma with loss of vascular anatomical relationships).

**Elastic fibre assessment**

Portal or periportal elastic fibres were not scored because a preliminary review showed that it was difficult to establish with certainty what constituted an excess of elastic fibres in the portal-periportal region in view of the presence of elastic fibres in normal portal tracts. In contrast, a bridging fibrous septum constituted a more defined pathological landmark for the assessment of the elastic fibre content.

In terms of septal elastic fibres, our initial review showed they ranged from delicate strands visible only at high magnification to dense compact bundles of strongly Victoria blue positive septa, or slender septa of similar intensity.

We chose the generic term of PCE and septal elastosis (rather than other possible terms such as pericellular or septal fibrillosis or microfibrillosis), regardless of the precise composition of elastic fibres in terms of oxytalan, elaunin and elastin (please see below) for sake of simplicity and to provide an immediate reference to elastic fibres.

**Interobserver variability of newly designed scoring systems**

The three experienced senior liver histopathologists in the authorship (TVL, JW, and AQ) scored independently 20 cases for PCE, septal elastosis, and fibrosis using the newly designed scoring systems mentioned earlier. Interobserver agreement was moderate for septal elastosis (κ = 0.60) and substantial for PCE (κ = 0.73), and fibrosis stage (κ = 0.62).

**Livers removed at transplantation for end stage ALD**

The most recent twenty livers removed at transplantation between 2018 and 2019 for end stage ALD were reviewed as detailed above. This cohort gave us the opportunity to compare the findings in our biopsies with those of end stage ALD, but also investigate possible changes in the pattern of elastosis related to the period of abstinence required for transplant eligibility.

**Control group**

This series consisted of 213 consecutive liver specimens on which the Victoria blue stain was carried out as part of the routine diagnostic assessment at the Royal Free Hospital laboratory service between 2018 and 2019. These included liver biopsies and the background liver of liver resection specimens or livers removed at transplantation for a broad range of liver disorders, as illustrated in table 2. The purpose of the control group was to test whether the deposition of pericellular fibres is relatively specific to ALD, which required a large series covering a broad range of pathologies.

**Mixed ALD sub-series to investigate elastic fibres morphology and composition.**

We selected twenty cases representing the full range of fibrosis and elastosis and including core needle biopsy and explant specimens. Sufficient tissue remaining in the paraffin block was an essential requirement in order to obtain 6 serial sections.

**Comparison between original and repeated Victoria blues stains.**

The repeated Victoria blue stains gave us also the opportunity to compare them to the original ones carried out routinely at the time of diagnosis. There was a good correlation overall between the original and repeated Victoria blue stains (including the oxidation step) as shown in Supplementary figure 2, and showing that the Victoria blue stain had maintained its strength over the years with no significant fading. In two instances the original Victoria blue was lighter in areas where delicate elastic (oxytalan-type) fibres were present, in comparison with the repeated Victoria blue stain (including the oxidation step), and more similar to the other repeated Victoria blue stain (without the oxidation step). This difference was probably due to variations in the technique used originally (e.g. variation in the duration of the oxidation step), but confirmed the presence of oxytalan-type fibres, and did not change the overall interpretation on septal elastosis.

**Statistics**

Frequency data was assessed using a chi-squared test with a critical alpha level of 0.05 for significance. This was used to investigate whether there was an association between the presence or absence of elastic fibres and other histological features. Interobserver variability was assessed by kappa analysis.

This study has been approved by the NRES Committee London -. Hampstead

(IRAS: 254793, REC Reference 07/Q0501/50)

Supplementary Figure 1

1a. Breakdown of overall diagnosis in study cohort of 180 biopsies from patients with ALD according to main histological diagnostic categories (NOS, steatosis, steatohepatitis). 1b. Breakdown of overall diagnosis as figure 1a according to fibrosis stage.


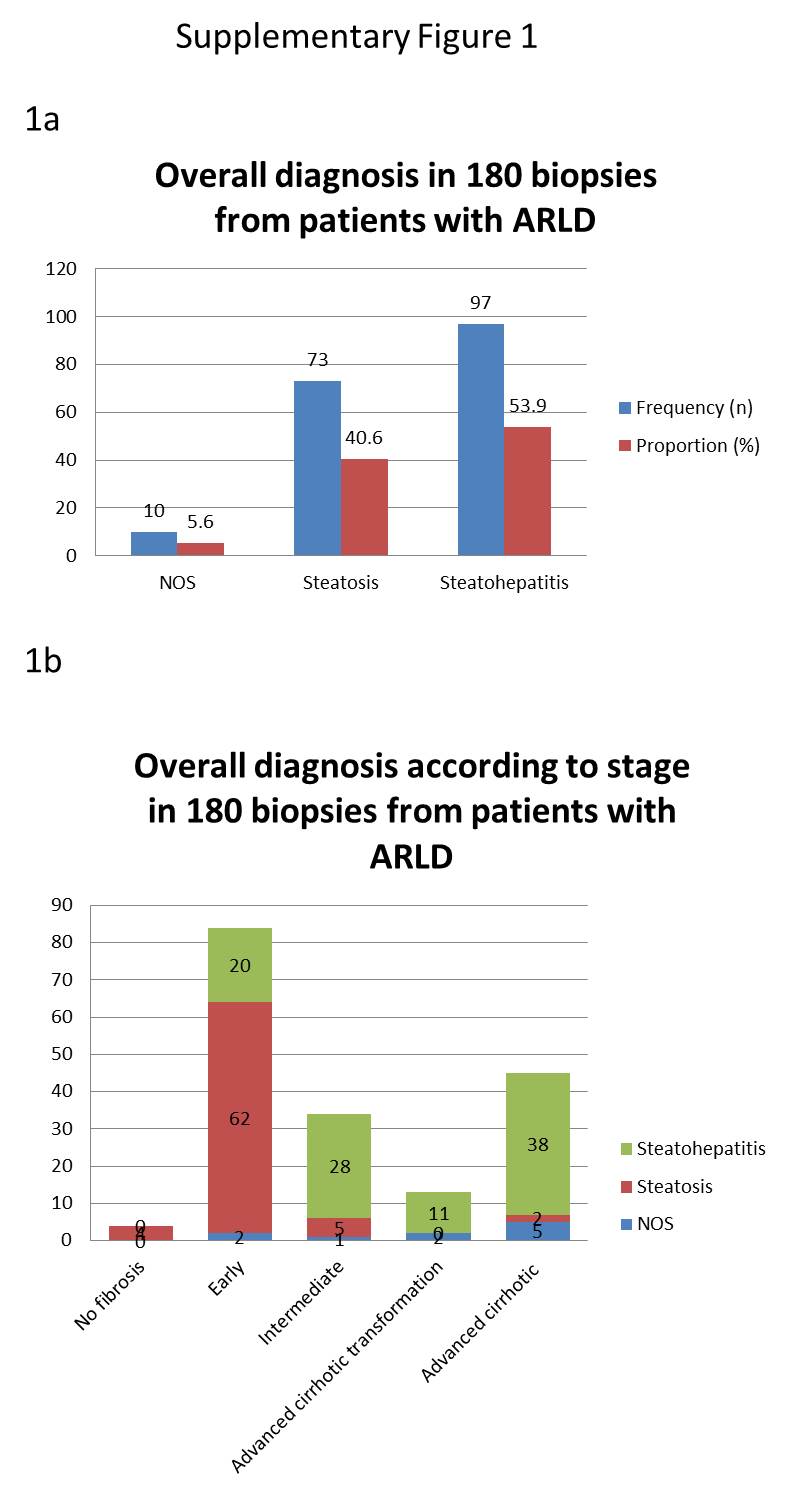


**Supplementary Figure 2**


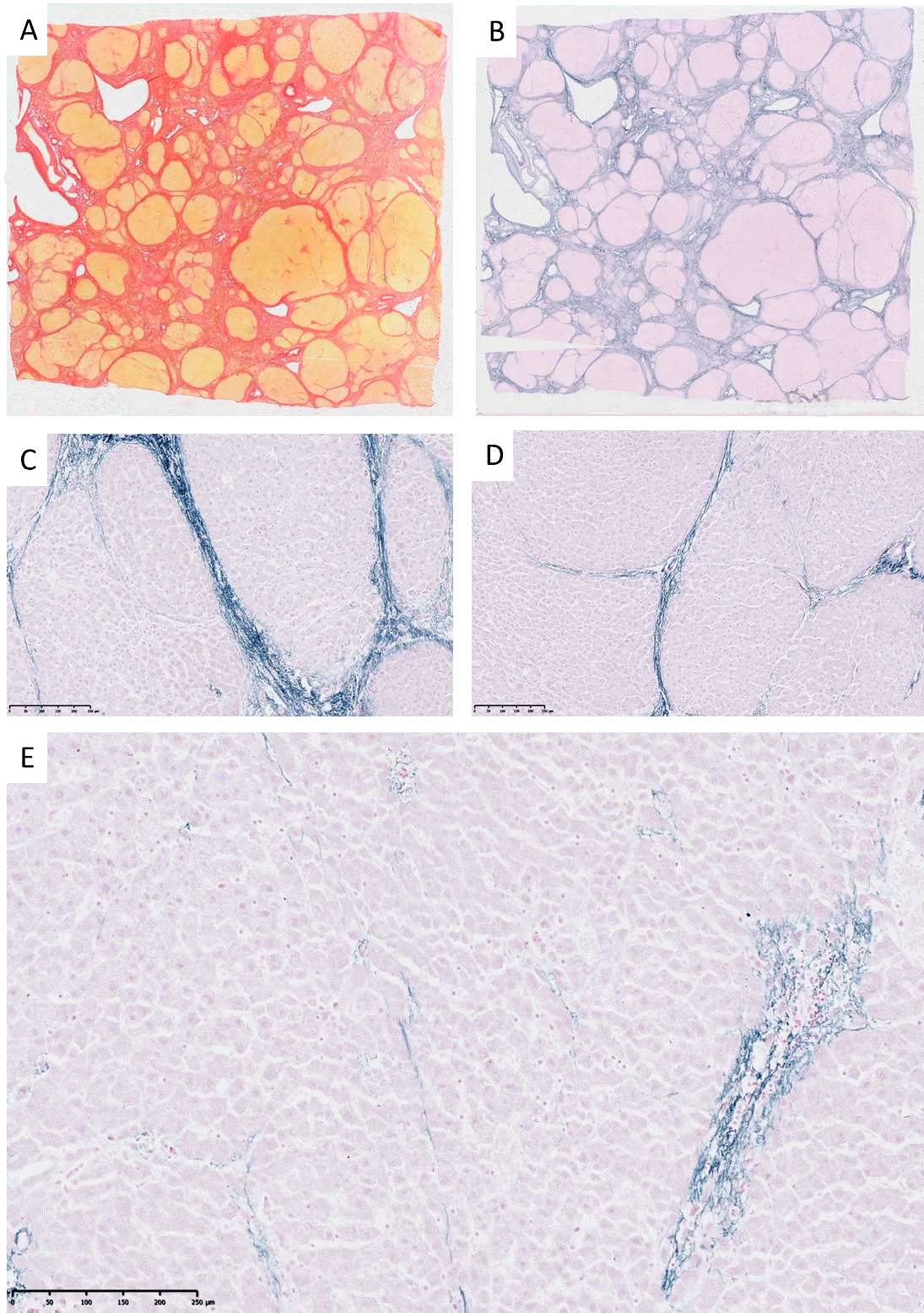


Supplementary figure 2. Liver removed at transplantation for end stage ALD. Advanced fibrosis and parenchymal nodules as evidenced with Picrosirius red stain (A). The matching Victoria blue stain shows easily visible elastic fibres at low magnification (B) mostly due to grades 3 (C) and 4 (D). Pericellular elastic fibres were often in the form of broken dense strands or in a more perisinusoidal distribution (E).

**Supplementary Figure 3**


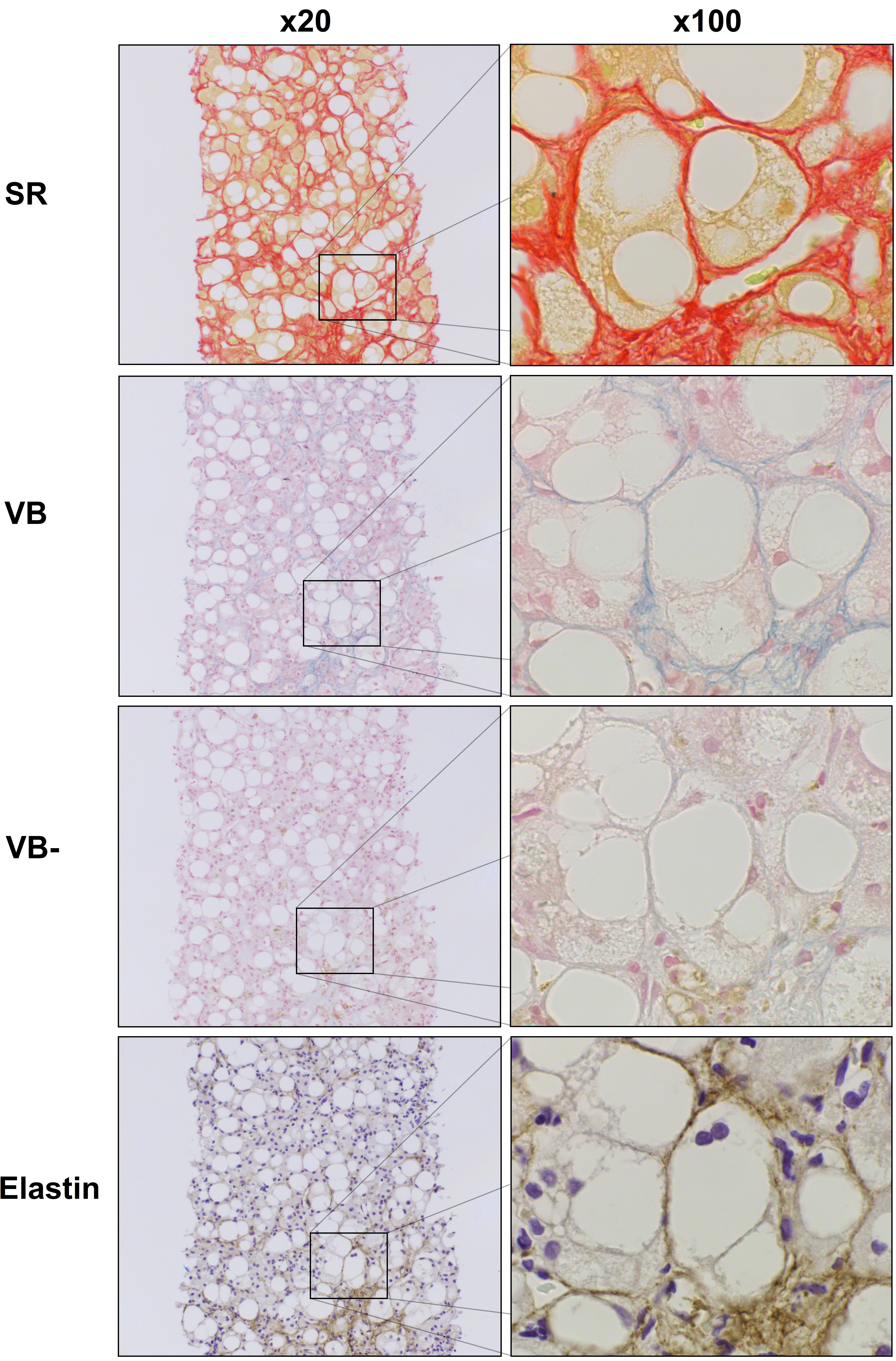


Supplementary Figure 3. Pericellular elastic fibres stain with the conventional VB stain but not with the modified Victoria blue method without oxidation step (VB-) indicating a considerable proportion of oxytalan fibres. Concomitant immunohistochemical elastin positive stain probably indicates that a proportion of these elastic fibres contain elastin probably in the form of elaunin.

Supplementary Figure 4


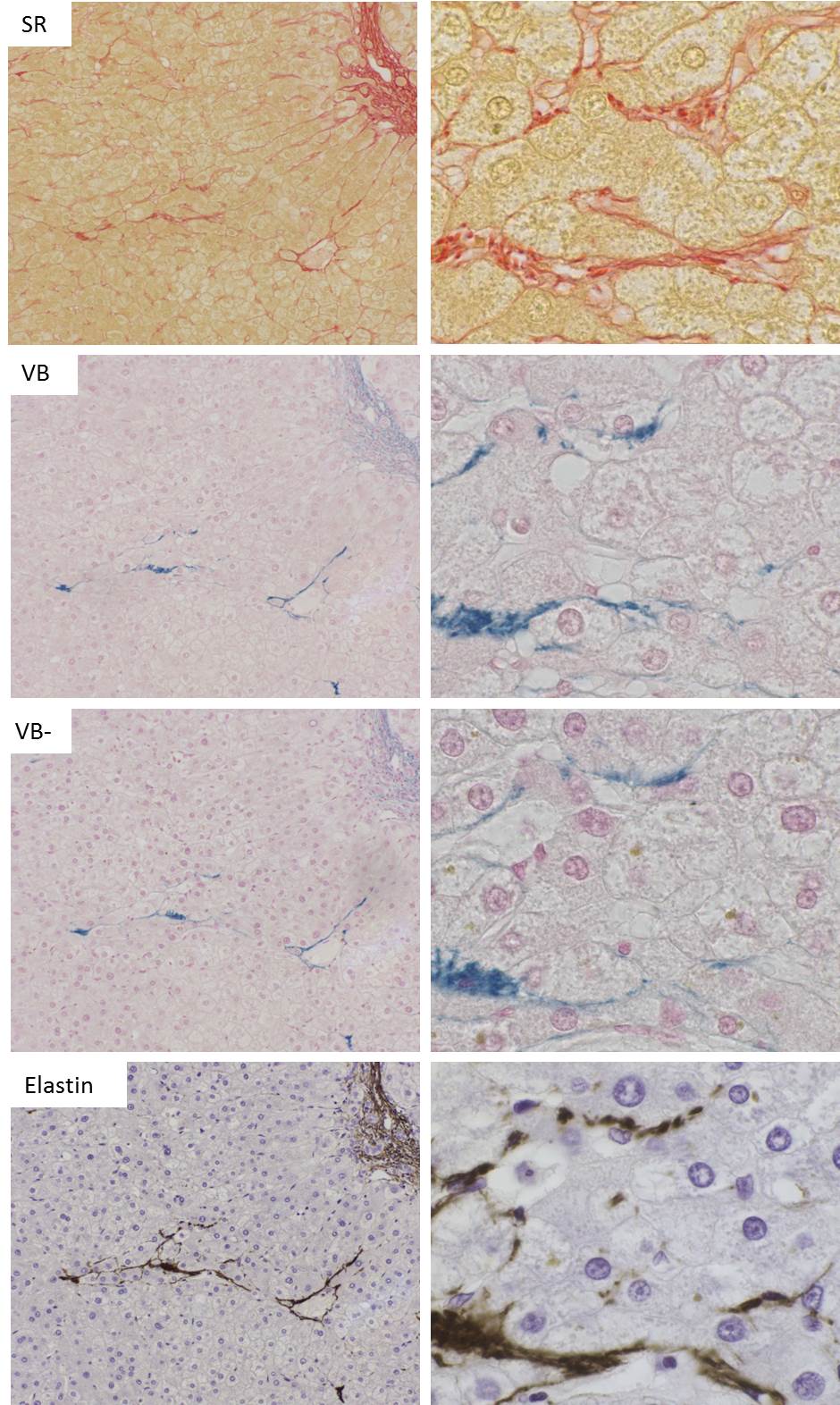


**Supplementary Figure 4.** Explant liver. Broken dense strands focally in a linear and more perisinusoidal than pericellular distribution (E) stain with both conventional and modified Victoria blue methods without appreciable reduction and probably composed of mature elastic fibres.

Supplementary table 1. Pericellular elastosis according to overall pattern of injury and fibrosis stage.

|  | NOS | | | | Steatosis | | | | Steatohepatitis | | | |
| --- | --- | --- | --- | --- | --- | --- | --- | --- | --- | --- | --- | --- |
|  | Total | None | Focal | PCE | Total | None | Focal | PCE | Total | None | Focal | PCE |
| No fibrosis | 0 | NA | NA | NA | 4 | 4 | 0 | 0 | 0 | 0 | 0 | 0 |
| Early | 2 | 1 | 1 | 0 | 62 | 37 | 21 | 4(6.9%) | 20 | 8 | 10 | 2(3.4%) |
| Intermediate | 1 | 0 | 1 | 0 | 5 | 1 | 2 | 2(3.4%) | 28 | 3 | 14 | 11(19% ) |
| Advanced cirrhotic transformation | 2 | 0 | 1 | 1 (1.7%) | 0 | 0 | 0 | 0 | 11 | 0 | 5 | 6(10.3%) |
| Advanced cirrhotic | 5 | 0 | 2 | 3(5.2%) | 2 | 0 | 1 | 1(1.7 %) | 38 | 0 | 10 | 28(48.3%) |
| Total | 10 | 1 | 5 | 4(6.9%) | 73 | 42 | 24 | 7(12.1%) | 97 | 11 | 39 | 47(81 %) |

**Supplementary table 2. Control group of 243 specimens to assess PCE across a broad range of liver disease.**

|  |  | Pericellular elastic fibres | | |
| --- | --- | --- | --- | --- |
|  |  | None | Focal | PCE |
| Autoimmune hepatitis | 12 (5.6%) | 9 (75%) | 2(16.7%) | 1(8.3%) |
| Biliary disease | 18 (8.5%) | 16 (88.9%) | 2(11.1%) | 0 |
| Liver in resection specimens for metastatic tumours | 23 (10.8%) | 21(91.3%) | 2(8.7%) | 0 |
| Other (miscellaneous) | 32(15%) | 23(71.9%) | 7(21.9%) | 2(6.3%) |
| Time 0 (baseline liver allograft biopsy) | 21(9.9%) | 14(66.7%) | 7(33.3%) | 0 |
| Post-OLT | 62(29.1%) | 48(77.4%) | 13(21%) | 1(1.6%) |
| Viral | 6 (2.8%) | 0 | 6(100%) | 0 |
| NASH | 30 (14.1%) | 7(23.3%)  CRN stage 1= 3  2=0  3=2  4=2 | 14(46.7%)  CRN stage 1= 4  2=2  3=6  4=2 | 9(30%)  CRN stage 1= 2  2=0  3=4  4=3 |
| Vascular | 9 (4.2%) | 3(33.3%) | 1(11.1%) | 5(55.6%) |
| **Total** | **213 (100%)** | **141**(66.2%) | 54(25.4%) | **18**(8.5%) |

**Supplementary table 3. Septal elastic fibres**

|  | **No of cases** | **Elastic fibres grade** | | | | |
| --- | --- | --- | --- | --- | --- | --- |
| **Fibrosis stage** |  | 0 | 1 | 2 | 3 | 4 |
| 2 (Intermediate) | 34 | 17 (18.4%) | 13(14.1%) | 3 (3.3%) | 0 | 1 (1.1%) |
| 3 (Advanced cirrhotic transformation) | 13 | 1 (1.1%) | 6 (6.5%) | 4 (4.3%) | 0 | 2 (2.1%) |
| 4 (Advanced cirrhotic) | 45 | 2 (2.2%) | 22 (24%) | 17 (18.5%) | 3 (3.3%) | 1(1.1%) |
| **Total** | **92** | **20 (21.7%)** | **41 (44.6%)** | **24 (26.1%)** | **3 (3.3%)** | **4 (4.3%)** |

Pattern 0: bridging fibrous septa with no elastic fibres;

Pattern 1: bridging fibrous septa with delicate fibres visible only at high magnification;

Pattern 2: elastic fibres visible at low magnification and clearly distinguishable from the residual portal or liver capsule elastic fibres;

Pattern 3: strong elastic bundles well visible at low magnification and of similar density, blending with or indistinguishable from the to the residual portal normal or liver capsule elastic fibres;

Pattern 4: slender, regressive type thin elastic bundles.

**Supplementary table 4. Change in elastic fibre stain with the modified Victoria blue method in comparison to the conventional Victoria blue stain, immunohistochemistry for elastin and fibrosis stage.**

|  |  |  | Septal elastic fibres | | | Pericellular elastic fibres | | | |
| --- | --- | --- | --- | --- | --- | --- | --- | --- | --- |
| Specimen | Fibrosis stage | Pattern of injury | Grade on conventional Victoria blue | Reduction in staining in the modified Victoria Blue | Elastin | Pericellular fibrosis (PSR) | Conventional Victoria blue | Reduction in staining in the modified Victoria Blue | Elastin |
| biopsy | 4 | Steatohepatitis | 2 | Partial | Diffuse | Diffuse | PCE | Partial | Diffuse |
| biopsy | 4 | Steatohepatitis | 2,4 | Marked, No reduction respectively | Diffuse | Patchy | Focal | Partial | Patchy |
| biopsy | 4 | Steatohepatitis | 2 | Marked | Diffuse | Diffuse | PCE | Marked | Patchy |
| biopsy | 4 | Steatosis | 3 | None | Diffuse | Patchy | PCE | None | Focal |
| biopsy | 3 | Steatohepatitis | 1 | Marked | Patchy | Diffuse | focal | Marked | Focal |
| biopsy | 2 | Steatosis | NA | Not applicable | NA | Diffuse | focal | Marked | Absent |
| biopsy | 3 | Steatosis | 1 | Marked | Diffuse | Patchy | Absent | Not applicable | Absent |
| biopsy | 4 | Steatohepatitis | 2 | Partial | Diffuse | Diffuse | focal | Marked | Focal |
| biopsy | 4 | Steatohepatitis | 2 | Partial | Diffuse | Diffuse | focal | Marked | Focal |
| biopsy | 4 | Steatohepatitis | 2 | Marked | Diffuse | Patchy | PCE | Partial | Patchy |
| biopsy | 4 | NOS | 2 | Minimal | Diffuse | Diffuse | PCE | None | Patchy |
| biopsy | 4 | Steatohepatitis | 2,4 | Partial, Minimal | Diffuse | Diffuse | PCE | Partial | Patchy |
| biopsy | 4 | Steatohepatitis | 1 | Partial | Patchy | Diffuse | PCE | Partial | Patchy |
| biopsy | 4 | Steatohepatitis | 1 | Marked | Patchy | Diffuse | PCE | Marked | Patchy |
| explant | 4 | NOS | 3,4 | None | Diffuse | Patchy | focal | None | Focal |
| explant | 4 | NOS | 3,4 | None | Diffuse | Patchy | focal | None | Focal |
| explant | 4 | NOS | 3,4 | None | Diffuse | Patchy | focal | None | Patchy |
| explant | 4 | NOS | 3,4 | none | Diffuse | Patchy | focal | None | Focal |
| explant | 4 | NOS | 3,4 | None | Diffuse | Patchy | focal | None | Focal |
| explant | 4 | NOS | 3,4 | None | Diffuse | Patchy | focal | None | Focal |

Supplementary references

1. Tiniakos D.G., Anstee Q.M., Burt A.D. Fatty Liver Disease. In *MacSween's Pathology of the Liver* (ed. Burt A.D., Ferrell L.D. and Hübscher S.G.)

308-371 (Elsevier, 2018).

2. Kleiner D.E., et al. Design and validation of a histological scoring system for nonalcoholic fatty liver disease. *Hepatology* **41**, 1313-21 (2005).

3. Ishak K., et al. Histological grading and staging of chronic hepatitis. *J. Hepatol.* **22**, 696-9 (1995).
